# Molecular survey of selected viruses in Pudus (*Pudu puda*) in Chile revealing first identification of caprine herpesvirus—2 (CpHV-2) in South American ungulates

**DOI:** 10.1080/01652176.2022.2149879

**Published:** 2022-12-28

**Authors:** Ezequiel Hidalgo-Hermoso, Sebastián Celis, Javier Cabello, Ignacio Kemec, Carolina Ortiz, Rocio Lagos, Juan Verasay, Dario Moreira-Arce, Pablo M. Vergara, Frank Vera, Fernando Esperón

**Affiliations:** a Fundacion Buin Zoo, Buin, Chile; b Departamento de Veterinaria, Parque Zoológico Buin Zoo, Buin, Chile; c Centro de conservación de la biodiversidad, Chiloé-Silvestre. Nal Bajo, Ancud, Chiloé, Chile; d Laboratorio Clínic, Hospital Veterinario SOS Buin Zoo, Buin, Chile; e Universidad de Santiago de Chile (USACH), Santiago, Chile; f School of Veterinary Medicine, Universidad San Sebastian, Puerto Montt, Chile; g Veterinary Department, School of Biomedical and Health Sciences, Universidad Europea de Madrid, Villaviciosa de Odón, Madrid, Spain

**Keywords:** Pudu, *Pudu puda*, caprine herpesvirus-2, pudu gammaherpesvirus 1, Chile

## Abstract

Viral diseases jeopardize the health of wildlife in Chile. However, this country lacks health surveillance programs that allow for defining preventive measures to tackle such diseases. The objective of this study was to determine the occurrence and the genetic diversity of pestivirus, herpesvirus and adenovirus in pudus from Chile. Blood samples from wild (n=34) and captive (n=32) pudus were collected between 2011 and 2019 and analyzed through consensus PCR. All the samples were negative to pestivirus and adenovirus. Herpesvirus was confirmed in four captive, and one wild pudu. All four zoo animals share the same sequence for both polymerase and glycoprotein genes. Both sequences share a 100% identity with caprine herpesvirus-2, classifying them in the same cluster as the Macavirus group. In turn, novel sequences of the polymerase and glycoprotein B genes were obtained from the wild pudu. Our study reports the first evidence of CpHV-2 infection in Chile and South American ungulate populations. Further research will be necessary to assess the pathogenicity of CpHV-2 in this species. It is also urgently recommended that molecular, serological and pathological screening should be conducted in Chilean wild and captive pudus to understand the impact of the herpesvirus on their populations.

## 1. Introduction

Viral diseases jeopardize the health of free and captive wildlife (Salgado et al. 2018; Hidalgo-Hermoso et al. 2020; Vergara-Wilson et al. 2021). Chile lacks official health surveillance programs for various diseases, which hinders the implementation of prevention and control measures for possible outbreaks. Herpesvirus, adenovirus and pestivirus represent three viral families that have been reported as causing several diseases in world cervid populations, including infectious keratoconjunctivitis, malignant catarrhal fever (MCF), bovine viral diarrhea, and adenovirus hemorrhagic disease (Passler et al. 2016; Sánchez Romano et al. 2018; Carvallo et al. 2020; Burek-Huntington et al. 2021; Dastjerdi et al. 2021), but little is known about their distribution and prevalence in wild ungulates in Chile.

The southern Pudu (*Pudu puda*), referred as Pudu in this study, is one of the world’s smallest deer that inhabits South American temperate forests. In Chile, Pudu populations are distributed throughout coastal and Andean temperate forests (36 °C–49 °C) (Pavez-Fox and Estay 2016). Pudus are mostly associated with well-conserved forests that comprise a dense understory (Eldridge et al. 1987; Meier and Merino 2007; Silva‐Rodríguez and Sieving 2012). Since deforestation and forest degradation have significantly affected Pudu distribution (Echeverria et al. 2006; Lara et al. 2012) the species has likely experienced a similar habitat reduction and population decline (Silva‐Rodríguez and Sieving 2012). Disturbed forests and commercial tree plantations are also inhabited by Pudus (Silva‐Rodríguez and Sieving 2012; Simonetti et al. 2013), which increases spatial co-occurrence between this species and domestic livestock, including cattle, goats, and sheep. As a consequence of this spatial interaction, the presence of pathogens from livestock has been documented in Pudus (Moreno-Beas et al. 2015; Salgado et al. 2018; Hidalgo-Hermoso et al. 2022) including exposure and infection by pestivirus (Salgado et al. 2018), and hence the importance of tracking cattle-related pathogens for Pudu survival (Silva-Rodríguez et al. 2021).

The Pudu is the most common species of South American cervids in European and North American zoos (Zims 2021). This fact, together with the lack of health surveillance programs for wildlife in their countries of origin, has the unintended result that most of the information about the diseases that affect this species originates from zoological institutions. The body of unpublished reports and personal communications with institutions working with captive and wild populations of Pudu in Chile, which describe clinical presentations compatible with viral infections, indicates an urgent need for screenings that confirm or rule out the presence of these diseases in the species under study.

The objective of this study is to confirm the occurrence and the genetic diversity of pestivirus, herpesvirus, and adenovirus in captive and free-ranging Pudus from Chile.

## 2. Materials and methods

### 2.1. Animal sampling

Blood samples from wild (*n* = 34) and captive (*n* = 32) Pudus were collected between 2011 and 2019 (Supplementary Table 1). Wild Pudu samples were collected on the day of admission to two wildlife rescue centers in Los Lagos region (USS: Universidad San Sebastian, Ch S: Chiloe Silvestre). Samples from captive-born Pudus were collected during clinical or preventive medicine procedures in two facilities, one in Region Metropolitana (BZ: Buin Zoo) and the other in Los Lagos (Ro: Romahue). Four BZ Pudus were sampled two times (I585, I586, I592, and I649). The protocol for Pudu sampling in the present study was approved by Veterinary Department of Buin Zoo and the Veterinary Heads of the wildlife rescue centers, and samples were collected by the veterinary staff of each institution. For blood sampling details, see the methodology described in Hidalgo-Hermoso et al. (Hidalgo-Hermoso et al. 2022).

### 2.2. Molecular detection and phylogenetic analysis

DNA and RNA were simultaneously extracted by a pressure filtration method (QuickGene® DNA tissue kit S, FujiFilm Lifescience, Tokyo, Japan), adding an RNA carrier in the lysate step, as previously described (Sacristán et al. 2015).

Adenoviral DNA detection was conducted respectively by means of the pan adenovirus PCR, previously described (Li et al. 2010). RT-PCR targeting a 288 bp fragment of the 5′ untranslated region (5′-UTR) of pestivirus was performed using primers specific to pestivirus (Vilcek et al. 1994).

Finally, samples were analyzed using a nested pan-PCR that amplified a fragment of approximately 215–315 bp of the HV DNA polymerase gene (VanDevanter et al. 1996). The obtained sequences were classified as gamma herpesviruses (see results). To obtain better robustness for sequence identification, a second nested PCR was performed to amplify a 500 bp fragment of the HV glycoprotein B gene for gamma herpesviruses (Ehlers et al. 2008). PCR primers and annealing temperatures for each PCR are detailed in Table 1.

**Table 1.** Primers used in the different PCR reactions.

Target	Sequence (5’-3’)	**Sense; location** ^a^	Annealing temperature	References
Adenovirus	CAGCCKCKGTTRTGYAGGGT	+, outer	48 °C	Li et al. (2010)
	GCHACCATYAGCTCCAACTC	-, outer		
	GGGCTCRTTRGTCCAGCA	+, inner	48 °C	
	TAYGACATCTGYGGCATGTA	-, inner		
Herpesvirus, polymerase	GAYTTYGCNAGYYTNTAYCC	+, outer	46 °C	VanDevanter et al. (1996)
	TCCTGGACAAGCAGCARNYSGCNMTNAA	-, outer		
	GTCTTGCTCACCAGNTCNACNCCYTT	-, outer		
	TGTAACTCGGTGTAYGGNTTYACNGGNGT	+, inner	46 °C	
	CACAGAGTCCGRTCNCCRTADAT	-, inner		
Herpesvirus, glycoprotein B^b^	CCTCCCAGGTTCARTWYGCMTAYGA	+, outer	46 °C	Ehlers et al. (2008)
	CCGTTGAGGTTCTGAGTGTARTARTTRTAYTC	-, outer		
	AAGATCAACCCCACIAGIGTIATG	+, inner	46 °C	
	GTGTAGTAGTTGTACTCCCTRAACATIGTYTC	-, inner		
Pestivirus	ATGCCCWGTAGGACTAGCA	+	50 °C	Vilcek et al. (1994)
	TCAACTCCATGTGCCATGTAC	–		

^a^ Outer PCR or inner PCR, only included for nested PCRs.

^b^ Only performed for molecular characterization of positive samples to herpesvirus detection.

Positive controls for adenovirus, pestivirus, and herpesvirus PCRs were included for each run of samples, and were, respectively: Canine adenovirus 1, Bovine viral diarrhea virus 2, and Canine herpesvirus 2 (Cabello et al. 2013).

Purified products from positive PCRs were sequenced by the Sanger method. In the case of the PCR which targets the polymerase gene of herpesviruses, the PCR products were sequenced with sequencing primers, TGVseq (5′-CATCTGATGTAACTCGGTGTA-3′) and IYGseq (5′-GACAAACACAGAGTCCGT-3′’), as previously reported (VanDevanter et al. 1996). For the PCR targeting the glycoprotein B gene, sequencing primers were the same that the used for the inner PCR.

Two sequences for each amplicon were obtained, one with the forward primer and the other one with the reverse. Both sequences were manually checked and alignment between them by ClustalW to obtain a consensus sequence. Consensus sequences were compared to those previously published in GenBank using a Blast search. Nucleotide (nt) and deduced amino acid (aa) p-distances were calculated with MEGA Software X after editing out the primers (Kumar et al. 2018). After ClustalW alignment of nt and aa sequences by MEGA software X (Kumar et al. 2018), nt and aa maximum likelihood phylogenetic threes were generated with 1000 bootstrap replicates for both polymerase and glycoprotein B genes.

### 2.3. Statistical analysis

The objective of statistical analysis was to explore differences between the prevalence of viral agents between captive and wild populations by calculating median values (www.winepi.net). A non-parametric test (Mann–Whitney U) was performed. Differences were considered statistically significant when *P* value was lower than 0.05. Statistics were performed by means of IBM SPSS vs 26.

## 3. Results

**Figure 1.**
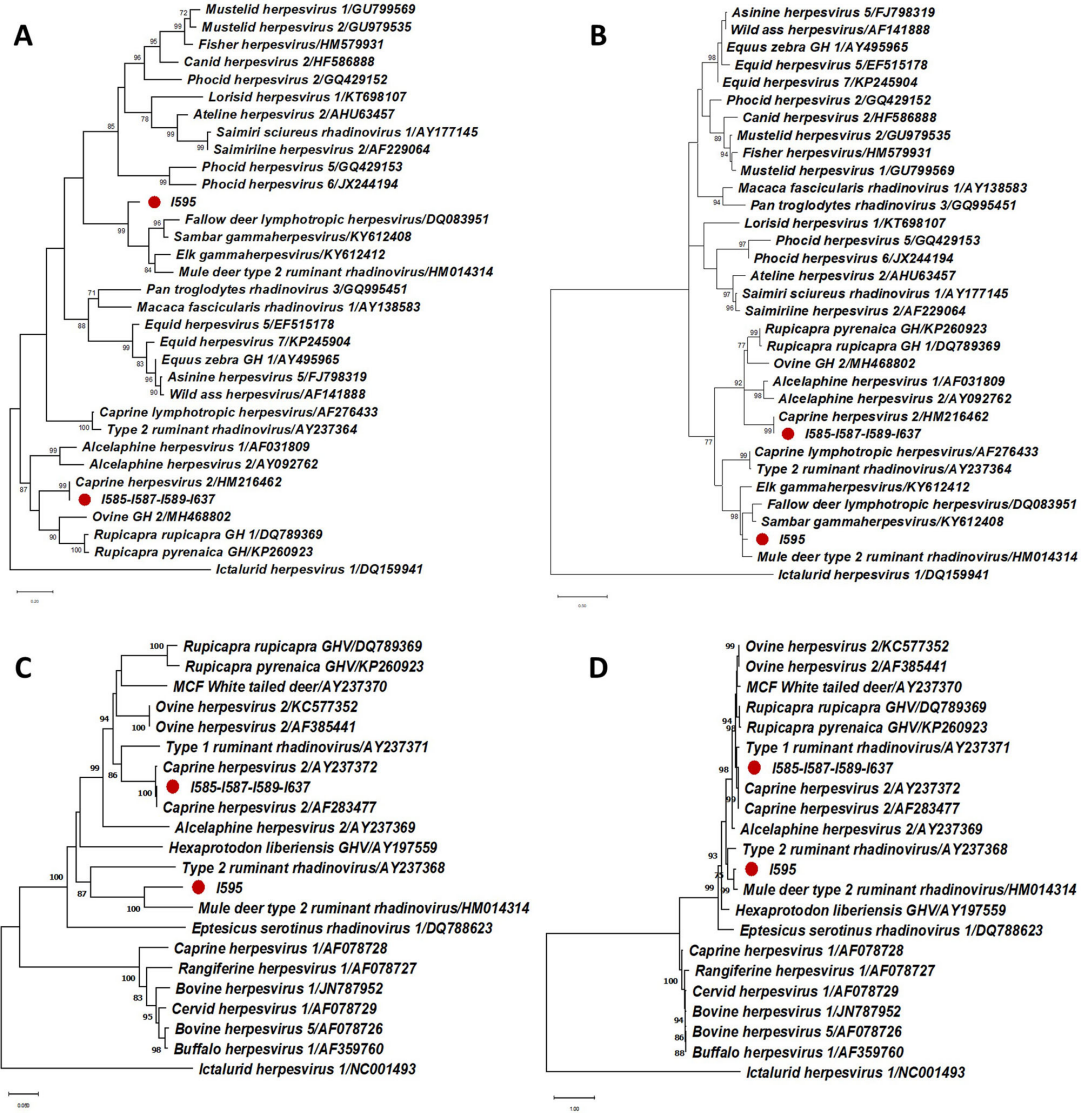
Maximum-likelihood phylogenetic trees of herpesviral sequences (A: polymerase gene, nucleotide sequences -160 bp, excluding primers-; B: polymerase gene, deduced amino acid sequences; C: glycoprotein gene, nucleotide sequences -420 bp, excluding primers-; D: glycoprotein gene, deduced aminoacid sequences). The reliability of the trees was tested with a bootstrap frequency of 1000 replicates. All bootstrap values smaller than 70% have been omitted. Each sequence has been expressed by the name of the virus as it is in the Genbank followed by the Genbank Accession Number. The Genbank Accession Number of the sequences obtained in this study are for polymerase gene: I585-I587-I589-I637 (LC667447) and I595 (LC667446); and for glycoprotein B gene: I585-I587-I589-I637 (LC667449) and I595 (LC667448). The Ictalurid herpesvirus is classified as an outgroup member.

All the samples were negative to pestivirus and adenovirus. Herpesvirus DNA was confirmed in five Pudus, four of them from captive populations (12.5% 95% CI: 1.0–24.0%) and the other one in wild populations (2.9% 95% CI: 0–8.6%). No statistically differences were found between wild and captive populations. All four zoo animals share the same sequence for both polymerase and glycoprotein genes. Both sequences have a 100% identity with caprine herpesvirus-2, classifying them in the same cluster as the Macavirus group (Figure 1). In turn, a novel sequence of the polymerase gene was obtained from the wild Pudu. This sequence has a 78.7% nucleotide identity with fallow deer lymphotropic herpesvirus as well as an 84.6% aminoacidic identity with Elk gammaherpesvirus and Mule deer type 2 ruminant rhadinovirus (Figure 1). Despite these low identities with the closest sequences the phylogenetic trees showed robust classifications (bootstrap frequency of 99% in the nucleotide tree and 98% in the deduced aminoacid tree) with the cluster formed by the Fallow deer lymphotropic herpesvirus (Genbank Accession Number DQ083951), Sambar gammaherpesvirus (Genbank Accession Number KY612408), Elk gammaherpesvirus (Genbank Accession Number KY612412), and Mule deer type 2 ruminant rhadinovirus (Genbank Accession Number HM014314). The glycoprotein B gene showed a nucleotide identity of 85.5% and an amino acid identity of 88.9% with Mule deer type 2 ruminant rhadinovirus (Figure 1). As a result of phylogenetic analysis, we could tentatively classify this novel virus as a ‘rhadinovirus-like’ of ruminants, with the name of ‘Pudu gammaherpesvirus 1’.

The sequences obtained in this study were assigned with the following Genbank Accession Numbers: Caprine herpesvirus 2, polymerase gene (LC667447), Caprine herpesvirus 2, and glycoprotein gene (LC667449); Pudu gammaherpesvirus 1 and polymerase gene (LC667446); Pudu gammaherpesvirus 1 and glycoprotein gene (LC667448).

## 4. Discussion

Our study confirms the useful application of molecular surveys to wildlife pathogens in regions like South America, where there is poor understanding of the susceptibility of such species to most livestock diseases (Navas-Suárez et al. 2018; Hidalgo-Hermoso et al. 2020; Sanchez-Vazquez et al. 2021). Gammaherpesvirus infection has been described in cervids worldwide (das Neves et al. 2020), but little is known about the gammaherpesviruses species infecting cervids in South America and ruminants in general in Chile. To the best of our knowledge, this is the first report of CpHV-2 in South America. According to the literature, there has been only one report of CpHV-2 infection in this species in which MCF affected a single Pudu at an Italian zoo (Modesto et al. 2015). Up to this date, a single MCF outbreak by OvHV-2 has been reported, in which 19 Sambar deers showing neurological symptoms died at a deer farm. In another case report, a single Brown brocket deer (*Mazama goazoubira*) died at a petting zoo in Brazil. Although this last case was not molecularly characterized (Driemeier et al. 2002), it is suspected that OvHV-2 was the cause, as it is the only MCF case reported in that country (Headley et al. 2020; Oliveira et al. 2021). A 20-year retrospective pathological study with samples of March deer (*Blastoceros dichotomus*) and brown brocket deer in Brazil reported findings of respiratory disease lesions with lymphoplasmacytic perivasculitis, in which a single Pudu from an Italian zoo developed signs of malignant catarrhal fever (MCF) (Navas-Suárez et al. 2018).

Viruses belonging to the MCF group have been described in at least 13 cervid species with variable susceptibility, with some species such as Pere David’s deer (*Elaphurus davidianus*), Red deer (*Cervus elaphus*), Sambar deer (*Cervus unicolor*), Sika deer (*Cervus nippon*), and White-tailed deer (*Odocoileus virginianus*) among the most susceptible to mortality by OvHV-2 (Crawford et al. 2002; Foyle et al. 2009; Li et al. 2013; Zhu et al. 2020). In contrast, CpHV-2 infections have been reported in six deer species, but some authors described that the virus is well adapted to this family (Zhu et al. 2020), because of the lack of acute infectious presentations. Although three of the four Pudus that tested positive for CpHV-2 died acutely between 1 and 3 days after sampling within this study, we lack scientific evidence to confirm that MCF generated by CpHV-2 was the cause of death.

The source of infection of the infected Pudus in this study is unclear, but it is hypothesized that aerial transmission occurred from a goat enclosure located 100 m from the Pudus. CpHV-2 aerial transmission *via* aerosol or wind has been reported among enclosures as far as 5 km apart (Li et al. 2008).

A low prevalence of herpesvirus in wild Pudus was detected, which suggests a limited exposure to the virus or a high rate of mortality. Studies about herpesvirus in wild cervids from Norway have found a high prevalence of infection: 48.6% in Reindeer (*Rangifer tarandus*) (das Neves et al. 2020) and 74% in Muskox (*Ovibos moschatus*) (Vikøren et al. 2013). However, since there is no information about the prevalence of these pathogens in domestic or wild ruminants in Chile, their epidemiological relevance at a regional level is unknown. The finding of a new sequence of gammaherpesvirus, tentatively Pudu gammaherpesvirus 1, has been reported in a wild Pudu without clinical signs related to the infection. Both polymerase and glycoprotein B sequences from this sample were classified in the same cluster of several gammaherpesvirus of deers, mostly belonging to rhadinoviruses. Rhadinovirus infections have been reported a few times in cervids; encephalitis has been observed in a free-ranging Sambar deer as the only associated pathology (Chang et al. 2018), while other studies have detected Rhadinovirus in cervids but without any pathological damage confirmed (McKillen et al. 2017; Patel et al. 2019). The most pathogenic viral strain recognized within this genus is the bovine gammaherpesvirus 4, which causes reproductive diseases in cattle, such as endometritis, vulvovaginitis and mastitis (Florencia et al. 2020) and has been molecularly detected in some European wild cervids (Kalman and Egyed 2005). However, there are no reports of pathologies in wildlife. This is thus the first report of a Rhadinovirus infecting a South American deer. It remains to be confirmed if this new virus is pathogenic to Pudu or other cervid species, or represents a health risk to livestock species.

No evidence of infection was confirmed for the other studied pathogens. Outbreaks of infectious diseases, with pathological evidence suggesting pestivirus or/and adenovirus etiology, were recently reported in wild Pudus justified the need to know the epidemiological status of these virus in Pudus in the region. Despite the relevance of adenovirus infection reported in cervids in other regions (Dastjerdi et al. 2021), no previous studies were conducted on the adenovirus family in Chilean deers, and the current evidence suggests a low prevalence or absence in Pudus. It is recommended to analyze tissue samples with molecular genetics tools, formalin-fixed, paraffin-embedded tissue by immunohistochemistry, and blood samples by serum virus neutralization assay, to confirm or rule out the presence of adenovirus in cervids in the region (Woods et al. 2018; Kauffman et al. 2021; Tomaszewski et al. 2021). Regarding pestivirus, previous reports of exposure and infection in wild and captive Pudu in Chile (Pizarro-Lucero et al. 2005; Salgado et al. 2018) suggest a very low prevalence in wild and/or epidemic episode in the zoo population in the most recent report. Serological screening in a large number of wild and captive animals is recommended for confirming or ruling out their epidemiological relevance in this species.

Our study reports the first evidence of CpHV-2 infection in Chile and South American ungulate populations. The findings about three of the infected Pudus point to the need of conducting a pathological study to confirm or rule out death by MCF. The previous reports of pathology by this virus in Pudus recommend including molecular screening of CpHV-2 in the differential diagnoses of deaths with clinical respiratory and/or gastrointestinal signs, or sudden death in Pudus at zoos and rehabilitation centers. Further research will be necessary to assess the pathogenicity of CpHV-2 in this species. It is also urgently recommended conducting molecular, serological, and pathological screening in Chilean wild and captive Pudus to understand the epidemiology of this virus and other herpesvirus, mainly in order to identify the source of infection in captive Pudus, and whether CpHV-2 has been involved in other infectious cases in wild and captive Pudu.

## Supplementary Material

Supplemental Material

## Data Availability

The original contributions presented in the study are included in the article/supplementary material; further inquiries can be directed to the corresponding author/s.
