# Supplementary material for: Molecular survey of selected viruses in Pudus (Pudu puda) in Chile revealing first identification of caprine herpesvirus—2 (CpHV-2) in South American ungulates
Source: Vet Q. 2022 Dec 28;43(1):1–7. doi: 10.1080/01652176.2022.2149879 (PMC9809401; doi:10.1080/01652176.2022.2149879)
Supplement: Supplemental Material [file TVEQ_A_2149879_SM0494.docx]

Supplementary Table 2. PCR results and sequences for herpesvirus from wild and captive Pudus (Pudu puda) in Chile.

| Animals | Date | Age | Origin | Nested pan-PCR | ntST |
| --- | --- | --- | --- | --- | --- |
| I649 | 10-11-2011 | Adult | BZ | Negative | - |
| I590 | 14-01-2013 | Adult | BZ | Negative | - |
| I591 | 14-01-2013 | Adult | BZ | Negative | - |
| I593 | 14-01-2013 | Adult | BZ | Negative | - |
| I649 | 14-01-2013 | Adult | BZ | Negative | - |
| I628 | 02-05-2013 | Adult | BZ | Negative | - |
| I588 | 17-12-2013 | Adult | BZ | Negative | - |
| I586 | 08-07-2015 | Adult | BZ | Negative | - |
| I585 | 27-07-2015 | Adult | BZ | Negative | - |
| I637 | 15-01-2017 | Adult | BZ | Positive | CpHV-2 |
| I589 | 20-01-2017 | Adult | BZ | Positive | CpHV-2 |
| I585 | 21-01-2017 | Adult | BZ | Positive | CpHV-2 |
| I586 | 21-07-2017 | Adult | BZ | Negative | - |
| I587 | 21-07-2017 | Adult | BZ | Positive | CpHV-2 |
| I594 | 02-08-2017 | Adult | BZ | Negative | - |
| I592 | 02-08-2017 | Adult | BZ | Negative | *-* |
| I592 | 18-04-2018 | Adult | BZ | Negative | *-* |
| I611 | 18-04-2018 | Adult | BZ | Negative | - |
| I612 | 18-04-2018 | Adult | BZ | Negative | - |
| I613 | 18-04-2018 | Adult | BZ | Negative | - |
| I614 | 18-04-2018 | Adult | BZ | Negative | - |
| I615 | 18-04-2018 | Adult | BZ | Negative | - |
| J541 | 03-06-2019 | Adult | BZ | Negative | - |
| J542 | 03-06-2019 | Fawn | BZ | Negative | *-* |
| J543 | 03-06-2019 | Fawn | BZ | Negative | - |
| J544 | 03-06-2019 | Fawn | BZ | Negative | - |
| I616 | 01-07-2017 | Adult | Ro | Negative | - |
| I618 | 01-07-2017 | Adult | Ro | Negative | - |
| I619 | 01-07-2017 | Adult | Ro | Negative | - |
| I620 | 01-07-2017 | Adult | Ro | Negative | - |
| I621 | 01-07-2017 | Adult | Ro | Negative | - |
| I623 | 01-07-2017 | Adult | Ro | Negative | - |
| I624 | 01-07-2017 | Adult | Ro | Negative | - |
| I625 | 01-07-2017 | Adult | Ro | Negative | - |
| I626 | 01-07-2017 | Adult | Ro | Negative | - |
| I627 | 01-07-2017 | Adult | Ro | Negative | - |
| H141 | 2012 | NDA | USS | Negative | - |
| H150 | 2012 | NDA | USS | Negative | - |
| H155 | 2012 | NDA | USS | Negative | - |
| H156 | 2012 | NDA | USS | Negative | - |
| H157 | 2012 | NDA | USS | Negative | - |
| H170 | 2012 | NDA | USS | Negative | - |
| H173 | 2012 | NDA | USS | Negative | - |
| I642 | 01-11-2015 | Adult | USS | Negative | - |
| I639 | 26-11-2015 | Fawn | USS | Negative | - |
| I641 | 02-12-2015 | Fawn | USS | Negative | - |
| I643 | 19-02-2016 | Adult | USS | Negative | - |
| I640 | 26-05-2016 | Fawn | USS | Negative | - |
| I606 | 23-09-2016 | Adult | USS | Negative | - |
| I608 | 15-11-2016 | Adult | USS | Negative | - |
| I607 | 07-03-2017 | Adult | USS | Negative | - |
| I636 | 29-03-2017 | Adult | USS | Negative | - |
| I600 | 05-07-2017 | Adult | USS | Negative | - |
| I601 | 10-07-2017 | Adult | USS | Negative | - |
| I595 | 03-08-2017 | Adult | USS | Positive | HV novel sequence |
| I602 | 09-08-2017 | Adult | USS | Negative | - |
| I598 | 31-10-2017 | Adult | USS | Negative | - |
| I599 | 20-11-2017 | Adult | USS | Negative | - |
| I603 | 12-02-2018 | Adult | USS | Negative | - |
| I604 | 18-04-2018 | Adult | USS | Negative | - |
| I605 | 02-05-2018 | Fawn | USS | Negative | - |
| I609 | 10-05-2018 | Adult | USS | Negative | - |
| I610 | 13-06-2018 | Adult | USS | Negative | - |
| J314 | 02-07-2018 | Adult | Ch S | Negative | *-* |
| J313 | 29-08-2018 | Fawn | Ch S | Negative | - |
| J528 | 16-01-2019 | Adult | USS | Negative | - |
| J529 | 08-03-2019 | Adult | USS | Negative | - |
| J530 | 20-03-2019 | Adult | USS | Negative | - |
| J531 | 29-04-2019 | Adult | USS | Negative | - |
| J540 | 09-05-2019 | Adult | USS | Negative | - |
|  |  |  |  |  |  |

NDA: no data available. ntST: nucleotide sequence types. USS: Universidad San Sebastian. Ch S: Chiloe Silvestre. BZ: Buin Zoo. Ro: Romahue.
